# Research on Innovative Apple Grading Technology Driven by Intelligent Vision and Machine Learning

**DOI:** 10.3390/foods14020258

**Published:** 2025-01-15

**Authors:** Bo Han, Jingjing Zhang, Rolla Almodfer, Yingchao Wang, Wei Sun, Tao Bai, Luan Dong, Wenjing Hou

**Affiliations:** 1 College of Computer and Information Engineering, Xinjiang Agricultural University, Urumqi 830052, China; 2 Engineering Research Center of Intelligent Agriculture Ministry of Education, Urumqi 830052, China; 3 Xinjiang Agricultural Informatization Engineering Technology Research Center, Urumqi 830052, China; 4 Department of Informatics, Fort Hays State University, Hays, KS 67601, USA; 5 School of Information Science and Engineering, Xinjiang College of Science & Technology, Korla 841000, China; 6 Agricultural Information Institute, Chinese Academy of Agricultural Sciences, Beijing 100080, China

**Keywords:** apple, quality grading, stem detection, image segmentation, artificial intelligence, machine learning, model compression, structural re-parameterization

## Abstract

In the domain of food science, apple grading holds significant research value and application potential. Currently, apple grading predominantly relies on manual methods, which present challenges such as low production efficiency and high subjectivity. This study marks the first integration of advanced computer vision, image processing, and machine learning technologies to design an innovative automated apple grading system. The system aims to reduce human interference and enhance grading efficiency and accuracy. A lightweight detection algorithm, FDNet-p, was developed to capture stem features, and a strategy for auxiliary positioning was designed for image acquisition. An improved DPC-AWKNN segmentation algorithm is proposed for segmenting the apple body. Image processing techniques are employed to extract apple features, such as color, shape, and diameter, culminating in the development of an intelligent apple grading model using the GBDT algorithm. Experimental results demonstrate that, in stem detection tasks, the lightweight FDNet-p model exhibits superior performance compared to various detection models, achieving an mAP@0.5 of 96.6%, with a GFLOPs of 3.4 and a model size of just 2.5 MB. In apple grading experiments, the GBDT grading model achieved the best comprehensive performance among classification models, with weighted Jacard Score, Precision, Recall, and F1 Score values of 0.9506, 0.9196, 0.9683, and 0.9513, respectively. The proposed stem detection and apple body classification models provide innovative solutions for detection and classification tasks in automated fruit grading, offering a comprehensive and replicable research framework for standardizing image processing and feature extraction for apples and similar spherical fruit bodies.

## 1. Introduction

The challenges posed by manual apple sorting, such as fruit collisions, surface dents, low grading efficiency, human influence, and significant labor consumption, are receiving growing attention [1]. Automatic apple grading [2] technology has been increasingly and widely implemented in the apple industry, serving as a crucial component in both the industry and market distribution. In this context, research has increasingly focused on the extraction of apple appearance features, non-destructive detection of the peel, and the design and optimization of fruit-grade classification models.

With the significant advancements in computer technology in the agricultural sector in recent years, these technologies have substantially improved the efficiency of apple production and have played a crucial role in apple grading. In the field of quality grading for Fuji apples, machine vision-based grading technology has evolved from using a single feature to employing multi-feature fusion techniques, leading to the development of numerous advantageous algorithms. In the extraction of a single feature like apple diameter, for instance, HayrettinT [3] employed the CIELab color space to extract apple masks and calculated the number of apple pixels in the x and y directions using the corresponding binary images. They used the pixel count to represent apple size. Sofu [4] measured the maximum diameter pixels both horizontally and vertically on four different sides of the same apple and combined these eight values to determine the apple’s diameter. Zhao [5] used a Laplacian operator to detect apple edges and locate the center point, ultimately calculating the maximum diameter of the apple. For the extraction of apple color features, Huang [6] used the RGB color space to segment an apple from its background via the G/B and R/G ratios. They applied a super-red and super-green segmentation method to divide red areas and calculate their size for color grading. Tan [7] converted apple images into the HIS color space, extracted the number of red pixels using the H component, and characterized the apples’ color features by the ratio of red pixels from front and rear views.

While using single features allows for quick apple grading, it cannot comprehensively reflect the quality grade of apples or meet diverse consumer demands. With further advancement in computer vision technology, researchers have gradually experimented with multi-feature grading of apples. Qiu [8] extracted color, texture, and shape features of defects, stems, calyxes, and apple surface areas; these features were used to train decision tree support vector machines for classifying defects vs. non-defects. Yu [9] utilized four images per apple to extract features like diameter, roundness, red area ratio, and defects, applying a weighted K-Means clustering algorithm for classification, achieving 93.3% accuracy. Employing multiple features in apple grading reduces misclassification and enhances the accuracy of the grading process.

With the rapid development of deep learning, the application of non-destructive testing and intelligent sorting technologies in the apple grading process has become increasingly widespread. Fan [10] combined color and diameter features to perform target detection and size grading on apple tree fruits, effectively identifying smaller targets and those with uneven lighting, achieving a grading accuracy of 90%. Shi [11] proposed a novel multi-view spatial network for apple grading tasks, incorporating apple size information as one of the grading criteria. Their approach starts with a well-pretrained lightweight CNN to extract low-level features of apples. Subsequently, a spatial feature aggregation module is constructed using bidirectional LSTM and mean pooling to explore relevant apple information from multiple perspectives, showing promising application prospects for the multi-view spatial network in apple grading tasks. Fan [12] also proposed a real-time apple defect detection method based on the YOLO V4 deep learning algorithm. The input image is synthesized from three consecutive infrared images. They simplified the YOLO V4 network using channel and layer pruning methods to increase detection speed. Additionally, they introduced an L1 norm-based non-maximum suppression (NMS) method to eliminate redundant prediction boxes after fine-tuning the pruned network, further enhancing the model’s detection speed.

Previous work has partially addressed the task of apple grading, primarily focusing on characteristics like color and size. However, the feature of apple stems has not been adequately studied, despite the fact that national apple grading standards require premium, first-grade, and second-grade apples to have intact stems. This highlights the stem characteristic as a crucial indicator, underscoring the need for an efficient and swift stem detection algorithm to enhance apple grading tasks. Additionally, complex background information can interfere with the accurate extraction of apple features, necessitating the development of an effective algorithm for fruit segmentation.

Building on prior research into automated apple classification and grading, this study uniquely integrates advanced computer vision techniques, image segmentation, and machine learning to create a comprehensive apple grading system. This system, aligned with apple grading standards, incorporates efficient and precise methods to evaluate the external quality of apples while avoiding secondary damage. The main contributions of this work are as follows:

1. We introduce, for the first time, a stem indicator in the apple grading task. By leveraging computer vision and state-of-the-art model compression techniques, we propose a high-precision, lightweight stem detection algorithm called FDNet-p. Extensive experiments demonstrate its superior performance. In the apple grading process, FDNet-p is initially applied to filter out apples without stems. Additionally, we propose an auxiliary positioning strategy to prevent premature or delayed triggering of the camera capture.
2. During feature extraction, we propose an improved DPC-AWKNN segmentation algorithm and incorporate an adaptive brightness equalization strategy to enhance fruit body segmentation. Image processing techniques are then employed to extract color, diameter, and shape features of apples, aligning these with real-world manual grading standards.
3. Based on the multi-feature information of apples, we use various performance metrics to comprehensively evaluate machine learning classification algorithms. Ultimately, the GBDT algorithm is selected for implementing intelligent apple grading.

The structure of the subsequent sections is as follows: In the Materials and Methods section, we first introduce the grading standards and dataset, then provide a detailed explanation of the principles of the FDNet-p stem detection algorithm, specific methods for apple feature extraction, and the GBDT apple grading model. In the Experiment and Result Analysis section, we compare and analyze the superiority of the FDNet-p and GBDT algorithms. The Discussion section outlines the limitations of this study, discussing potential future work. Finally, the Conclusions section summarizes our research and outlines future research directions.

## 2. Materials and Methods

### 2.1. Grading Standards and Dataset Construction

#### 2.1.1. Apple Grading Standards

According to the national standard for fresh apples [13], regarding the classification requirements for different types of apples, we extracted four key indicators: stem features, color features, diameter features, and shape features. We also revised a streamlined apple grading standard by integrating the local grading standards for Fuji apples. The stem feature strictly adheres to the national standard, requiring all three different grades of apples to have stems. Based on this, we further refined the diameter and shape features in conjunction with local standards. According to the national standard, the diameter feature for premium and first-grade apples is set at 70 mm or more; in our experiments, we set the diameter feature for premium apples at 80 mm or more. Additionally, we provided a detailed classification for shape features, with the specific grading standards outlined in Table 1. In the grading task, the priority of the four features is as follows: stem, color, diameter, and shape. If the relevant features do not meet the requirements for a particular grade, the apple is directly classified into the next lower grade.

#### 2.1.2. Apple Stem Dataset Construction

**Figure 1:**
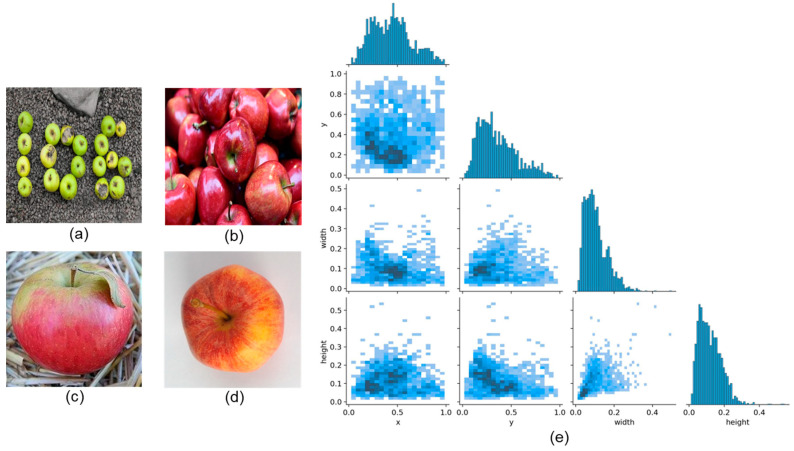
Dataset samples and visualization. (**a**) Complex background + multiple targets + small size. (**b**) Simple background + multiple targets + normal size. (**c**) Complex background + single target + normal size. (**d**) Simple background + single target + normal size. (**e**) Detailed distribution of annotation boxes.

For the apple stem detection task, there is currently a lack of publicly available datasets. To address this, we constructed a high-quality image dataset named Apple_Stem using both web scraping and manual collection methods. Initially, web scraping technology was utilized to acquire clear images of apples from the internet. To enrich the dataset’s diversity, we also incorporated apple images from complex scenarios, including multi-target and small-target stem images across various settings, enhancing the model’s robustness against interference. Additionally, we randomly augmented some sections of the dataset by applying image rotation techniques (15°, 45°, and 60°) to apples with stem targets on complex backgrounds, based on ideal conditions in automated grading pipelines. This augmentation allowed the model to effectively learn the various poses of apples in grading lines, thus improving model robustness and generalization. Ultimately, the dataset comprised 1530 clear images, divided into training, validation, and test sets at a ratio of 8:1:1. We employed the X-AnyLabeling 2.3.0 annotation tool for manual labeling of apple stems. Figure 1 illustrates specific data samples and the visualization of data distribution, in Figure 1e, where the center point coordinates, x and y, of the annotation box are proportional to the original image’s width and height and the annotation box’s width and height follow the same proportions. The varying shades of blue indicate the distribution of their quantities.

#### 2.1.3. Construction of an Apple Grading Dataset

In the research on apple grading models, we utilized an advanced image acquisition device equipped with an industrial-grade 3206IR-CUT high-definition distortion-free camera. This camera features an advanced CMOS sensor (1/2.7 inch) and operates at a frame rate of 30 frames per second, ensuring seamless image capture of apples. The device is fitted with an IR-CUT function to correct color deviation and enhance brightness in low-light environments. In well-lit conditions, the IR-cut filter is activated, allowing the CMOS sensor to capture the true colors of apples. When the sensor detects insufficient light, an automatic LED infrared fill light (invisible to the naked eye) is activated and the IR-cut filter is removed, enabling the infrared filter to function. This helps the camera make full use of LED infrared light, significantly improving image capture performance in low-light settings, ensuring the clarity and accuracy of apple images. The industrial camera captures three different angles of each apple, and the images are sent to a local computer for storage. Our chosen image acquisition equipment ensures stable and accurate image capture under varying lighting conditions, meeting the consistency and high reliability required in industrial applications. Our study used 126 apples as the research subjects for developing a classification model. Images of the apples were taken from the top, front, and rear views to extract three features: color, diameter, and shape.

### 2.2. Apple Grading Overall Design

**Figure 2:**
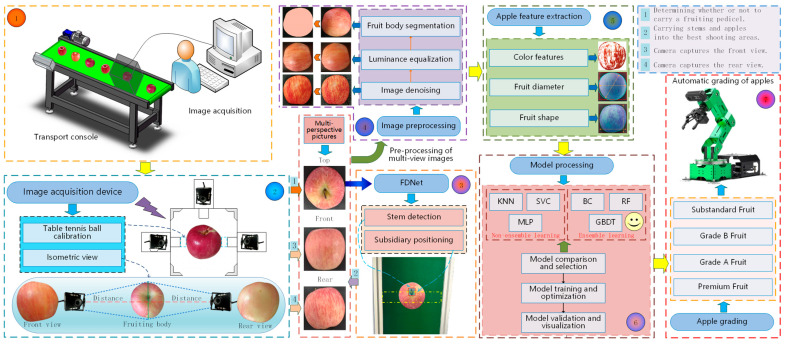
Schematic diagram of apple grading.

This study developed a simplified apple grading pipeline apparatus, primarily comprising seven components: a conveyance control system, an image acquisition unit, stem detection, fruit body image preprocessing, multi-feature extraction, grading model evaluation, and automatic sorting. By effectively integrating traditional machine learning techniques with cutting-edge deep learning technologies, a new architecture was designed specifically for the apple grading task, achieving efficient grading of apples. The detailed structure is illustrated in Figure 2.

The conveyance control system consists of a standard conveyor belt and a drive motor, which can dynamically adjust the speed of the pipeline according to requirements. The image acquisition unit includes a high-performance computer, an image acquisition device, and high-definition cameras. Inside the image acquisition unit, three high-definition wide-angle distortion-free industrial cameras are placed. During image capture, a ping-pong-ball-assisted calibration method is used first. By calculating the error between the diameter of the ping-pong ball in the captured image and its actual diameter, the distance between the camera and the apple is determined. When an apple is detected within the top camera’s field of view, the camera in the overhead image capture area checks for the presence of a stem. Apples without stems will not trigger the capture of frontal and rear views and will not proceed to the subsequent grading process. This enhances the efficiency of the apple grading pipeline and reduces resource consumption to some extent. The stem is also used for auxiliary positioning, and once the stem detection box fully enters a designated area, apples with stems trigger the computer to control the other two cameras to capture the front and rear views in sequence. The front and rear image capture focuses on the apple’s maximum cross-sectional area, approximating the apple as a sphere. High-definition cameras use an equidistant view method to capture the entire side view of the apple from the front and rear angles. Image processing operations are then applied to extract key feature information of the apples, followed by model comparison, training, and evaluation. The model with the optimal performance is selected to determine the apple’s grade. Finally, a robotic arm sorts the apples into specified grade areas.

### 2.3. Stem Detection and Fruit Body Subsidiary Positioning

**Figure 3:**
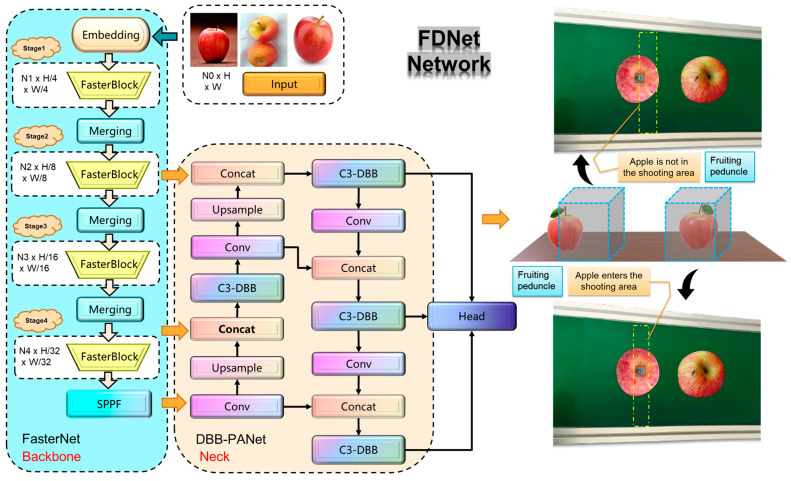
FDNet network structure and subsidiary positioning.

According to national and local standards, the presence of a stem is a key factor in distinguishing graded apples from non-graded ones. To effectively filter out apples missing stems, a lightweight stem detection algorithm must be designed. This prevents these apples from entering subsequent grading processes, further improving the efficiency of the apple grading pipeline and reducing unnecessary resource consumption. Another task for stem detection is to assist in positioning the fruit body, ensuring that the front and rear cameras are triggered correctly, avoiding premature or delayed activation that could prevent accurate front and rear image capture of the apple. During image acquisition, the stem detection bounding box is integrated with the camera triggering mechanism. When the stem is fully within the predefined area, the camera captures the apple, ensuring that clear, multi-view images are obtained. For the task of stem detection, this paper draws on the YOLOv5s [14] object detection algorithm architecture to propose a more advanced lightweight stem detection algorithm (FDNet). As shown in Figure 3, FDNet employs an efficient FasterNet [15] combined with SPPF modules for feature extraction, reducing model complexity and optimizing memory access strategies to minimize redundant computations and lower resource consumption. A multi-scale structural re-parameterization feature fusion network (DBB-PANet) was designed to enhance the model’s ability to detect stems of varying sizes. Additionally, a model compression strategy based on LAMP [16] scores was introduced, resulting in an extremely lightweight stem detection algorithm.

#### 2.3.1. FasterNet Feature Extraction Network

**Figure 4:**
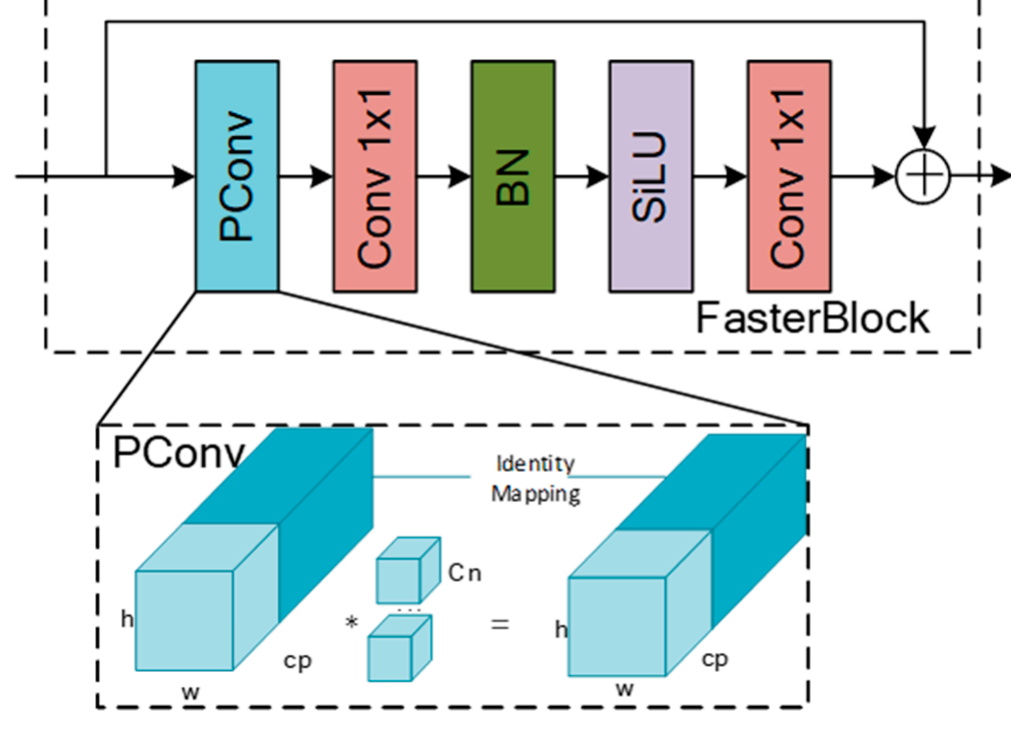
FasterBlock module structure. * means operation.

For the task of stem detection, there is a problem of high redundancy due to the similarity between feature maps. Moreover, in the apple grading pipeline, terminal devices have limited resources. To reduce the computational load and complexity of the model, this paper redesigns the feature extraction network using the efficient FasterNet model. The FasterNet network has three lightweight architectures: t0, t1, and t2. This study opted for the smallest model size, FasterNet-t0, with the specific network structure shown in the Backbone section of Figure 3. It includes four stages and consists of three modules: Embedding, Merging, and FasterBlock. These modules extract visual feature information of stems at the network’s shallow layers and semantic feature information at deeper layers. The Embedding and Merging modules are used to transform the dimensions and sizes of feature maps. The core FasterBlock module was constructed based on the Partial Convolution (PConv) operator, as illustrated in Figure 4. PConv performs feature extraction only on local feature maps, while the remaining channels retain the original feature information. This reduces computational load and memory access while decreasing redundancy in feature information. The Pointwise Convolution (PWConv) operator is used to fully integrate cross-channel feature information, enhancing diversity, and a residual connection was introduced to prevent gradient vanishing.

#### 2.3.2. DBB-PANet Feature Fusion Network

**Figure 5:**
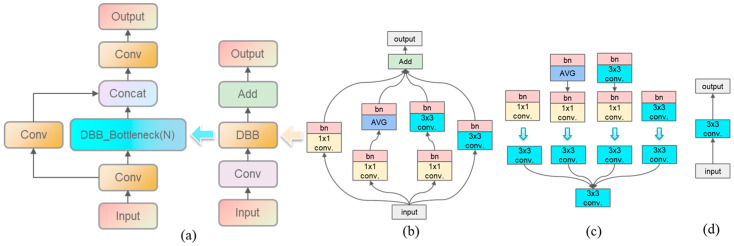
C3-DBB module. (**a**) Detailed structure of the C3-DBB module. (**b**) Structure of the DBB module during the training phase. (**c**) Re-parameterization operation of the DBB module. (**d**) Structure of the DBB module during the inference phase.

The size and shape of fruit stems vary, and the C3 module in the YOLOv5s neck network (PANet) is a single-branch structure. It often focuses more on feature information within a single locality during feature fusion, which results in poor capability for capturing information of different-sized objects. To address this issue, this paper introduces a multi-scale approach and structural re-parameterization technique to innovate the C3 module. The C3 module is restructured with a DBB [17] module, resulting in a more competitive C3-DBB feature fusion module, as shown in Figure 5a. This approach effectively captures feature information of stems of various sizes. The core component, the DBB module during the training phase, is illustrated in Figure 5b.

In the training phase, the C3-DBB module uses a multi-scale approach to capture rich feature information. It employs four different strategies to extract feature information: (I) 1 × 1 conv-bn, (II) 1 × 1 conv-bn + AVG-bn, (III) 1 × 1 conv-bn + 3 × 3 conv-bn, and (IV) 3 × 3 conv-bn. During the inference phase, structural re-parameterization is used to merge the four different multi-branch structures into a single-branch structure. Among these, strategies (2) and (3) require merging within the branches first. Assuming that two inputs are $F^{\left(1\right)}\in {\mathbb{R}}^{D_{1}\times C\times 1\times 1},b^{\left(1\right)}\in {\mathbb{R}}^{D_{1}}$ and $F^{\left(2\right)}\in {\mathbb{R}}^{D_{2}\times C\times K\times K},b^{\left(2\right)}\in {\mathbb{R}}^{D_{2}}$, the output is as shown in Equation (1):

(1) $$O^{\prime }=(Input⊙F^{\left(1\right)}+REP(b^{\left(1\right)}))⊙F^{\left(2\right)}+REP(b^{\left(2\right)})$$

In this context, ⊙ represents the convolution operation, while REP(⋅) denotes the padding operation applied to the batch normalization (bn) layer.

Since $F^{\left(1\right)}$ represents the 1 × 1 convolution, a linear recombination strategy is used to merge the parameters into the convolution kernel of $F^{\left(2\right)}$, as shown in Equation (2):

(2) $$F^{\prime }\leftarrow F^{\left(2\right)}⊙T(F^{\left(1\right)}),T(F^{\left(1\right)})\in {\mathbb{R}}^{C\times D_{1}\times 1\times 1}$$

In this context, T denotes the dimension transformation operation and the symbol “<-” indicates the transformation process and has the same meaning in the following text.

To construct the partial bias term, $\overset{\wedge }{b}$, it is represented as shown in Equation (3):

(3) $$\hat{b}\leftarrow \sum_{d=1}^{D}\sum_{m=1}^{K}\sum_{n=1}^{K}b^{\left(1\right)}F_{j,d,m,n}^{\left(2\right)},1\leq j\leq D_{2}$$

Merge all bias terms as shown in Equation (4):

(4) $$b^{\prime }\leftarrow \hat{b}+b^{\left(2\right)}$$

Finally, merge within the branch to form a new convolution operation, as shown in Equation (5):

(5) $$Concat(\cdot )=Input⊙F^{\prime }+REP(b^{\prime })$$

The merging methods among the four branches align with the strategy used in RepVGG [18]. The specific diagram of branch merging is illustrated in Figure 5c. The DBB module reduces the model’s parameter count without losing accuracy, and the structure after structural re-parameterization is shown in Figure 5d. By utilizing the C3-DBB module, the feature fusion network (PANet) was improved, leading to the proposal of the DBB-PANet network to enhance the feature fusion capability of the model.

#### 2.3.3. Model Lightweighting

In the task of stem recognition, the FDNet detection algorithm meets the requirements for detection accuracy but requires substantial computational power and memory to execute the model inference process. Additionally, there are limited device resources in the apple sorting pipeline. Therefore, this work employs a model compression technique based on LAMP (Layer-Adaptive Magnitude-Based Pruning) scores to reduce the parameters and computations of the FDNet model, achieving lightweighting and presenting an ultra-lightweight FDNet-p stem detection model that meets the deployment needs of edge devices in sorting pipeline operations. Channel pruning based on LAMP scores is an adaptive structured pruning technique at the channel level. It involves calculating the L2 norm of the weights of each input channel, sorting them, and establishing index mapping based on the newly generated sequence to remove weights and smaller channels, thereby achieving model compression. Channel pruning based on LAMP scores does not introduce additional computations or parameters and automatically selects inter-channel sparsity for pruning. The definition of the LAMP score is shown in Equation (6).

(6) $$LAMP_{Score}(u;C_{W}):=\frac{{\left(C_{W}(u)\right)}^{2}}{\sum_{u\leq v}{\left(C_{W}(v)\right)}^{2}}$$

In this context, the symbol “:=“ represents the definition of the LAMP score, u represents the target channel, ${\left(C_{W}(u)\right)}^{2}$ denotes the sum of squares of the weights of the target channel, and $\sum_{u\leq v}{\left(C_{W}(v)\right)}^{2}$ indicates the sum of squares of the weights of all remaining channels that have not been pruned and have indices greater than the target channel index.

### 2.4. Apple Feature Extraction

**Figure 6:**
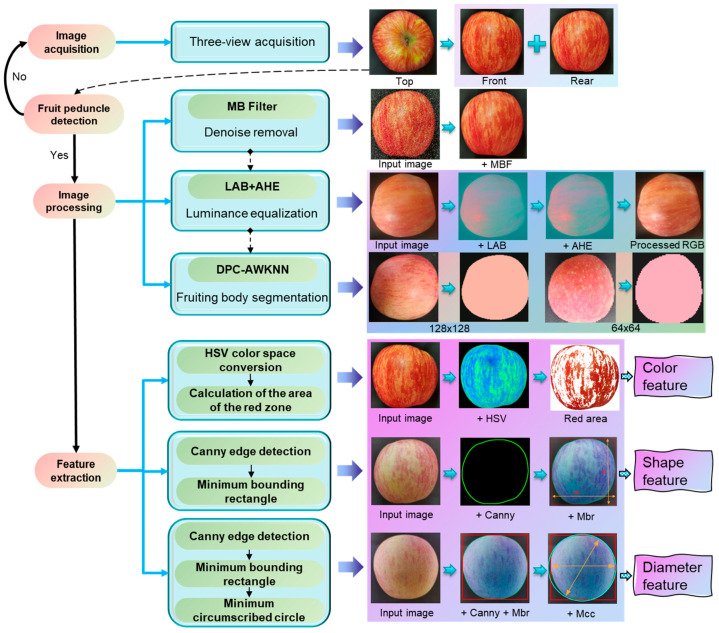
Apple feature extraction scheme.

In the image acquisition system, we use a top camera to capture an overhead view and employ the FDNet-p algorithm to detect apple stems. When an apple reaches the optimal shooting area, the other two cameras are triggered to capture the front and rear views of the apple. The images from these three perspectives are then batch-processed using image processing methods. During the apple feature extraction phase, we first suppress the impact of noise in the foreground image, then perform brightness equalization, segment the apple’s contour, and finally extract three important features: color, diameter, and shape. The specific operation process is shown in Figure 6.

#### 2.4.1. Noise Processing

When high-definition cameras capture images of apples, they ensure the clarity of apple details but are also susceptible to minor noise from the natural environment, which can hinder the subsequent extraction of apple features. To address this issue, this paper proposes a noise processing method that combines median filtering [19] and bilateral filtering [20]; its name is the MB Filter. The MB Filter algorithm rearranges each pixel and its neighborhood in the image, selects the median pixel for replacement, and combines it with a Gaussian-weighted filtering method in both the spatial and color domains, as described in Equations (7) and (8).

(7) $$I_{med}\;(p)=med(\{I(q)|q\in N(p)\})$$

(8) $$G_{med}\;(p)=\frac{1}{W_{p}}\sum_{q\in N(p)}I_{med}\;(q)\cdot f_{s}\;(∥p-q∥)\cdot f_{r}\;(∥I_{med}\;(p)-I_{med}\;(q)∥)$$

where $I_{med}$ and $G_{med}$ represent the median filter and the bilateral filter, $f_{s}$ and $f_{r}$ denote the Gaussian functions in the spatial and color domains, $W_{p}$ is the normalization factor, and N(p) indicates the neighborhood of pixel p.

The MB Filter algorithm first uses median filtering to suppress noise to the greatest extent possible and then applies bilateral filtering to adaptively smooth the noise regions in the image. The further processes ensure that the apple’s contours remain clear and that important detail information is not lost.

#### 2.4.2. Apple Segmentation

**Figure 7:**
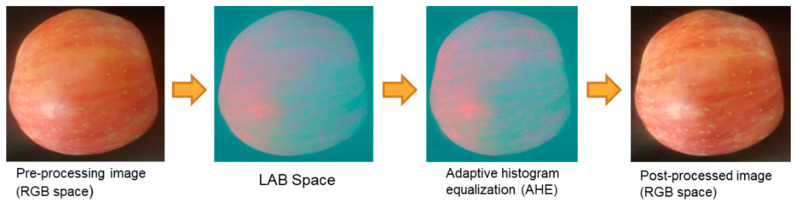
Brightness equalization algorithm processing procedure.

The background information significantly impacts the results of apple feature extraction, making it necessary to precisely segment the fruit from the background. To ensure more accurate apple segmentation, this paper designed a preprocessing algorithm tailored for the task scenario of an apple grading pipeline, addressing the issue of uneven brightness on apple surfaces. Initially, the image in the RGB space is converted to the LAB space, where the L component representing brightness is separated. Adaptive histogram equalization [21] is then applied for brightness processing. Subsequently, the processed L component is merged with the A and B components. Finally, the image in the LAB space is converted back to the RGB space. As shown in Figure 7, the processed image exhibits a more uniform surface brightness, partially resolving the issue of uneven illumination on the apple surface captured by the camera.

The preprocessed apples are segmented using a clustering algorithm. The Density Peak Clustering (DPC) [22] algorithm is a density-based clustering method that can automatically determine cluster centers and the number of clusters. However, its local density calculation for sample points is based on a cutoff distance, which does not account for the distribution of samples within the neighborhood. The choice of cutoff distance significantly affects the apple segmentation results. To address this issue, this paper proposes an improved DPC clustering algorithm (DPC-AWKNN) for apple segmentation. This method employs the Adaptive Weighted K-Nearest Neighbors (AWKNN) approach to adaptively select the nearest neighbors for calculating weighted local density, thereby eliminating the reliance on a global distance threshold. It automatically adjusts to the local density of points, effectively identifying density peaks. This enables the DPC algorithm to dynamically handle apple images with varying densities and shapes, reducing its sensitivity to the cutoff distance parameter. The specific computational process of DPC-AWKNN in the apple image segmentation task is outlined in Algorithm 1. Initially, the Euclidean distance [23] from each pixel to other points is calculated. Then, the AWKNN algorithm is used to compute the weighted local density and determine the minimum distance. Finally, the cluster centers are identified, and the class labels for the remaining pixels are assigned, achieving segmentation of apples from the background.
**Algorithm 1: DPC-AWKNN**Input: Image I Output: Cluster labels 1:Compute the distance matrix (D) for each pixel point.For each pixel point (i, j): D[i][j] = Euclidean distance (i, j). 2:Compute local density (ρ) using Adaptive Weighted K-Nearest Neighbors.For each pixel point i:  ρ[i] = 1/(average distance to K-Nearest Neighbors).3:Compute minimum distance (δ) to a higher density point.For each pixel point i:  If ρ[i] is the highest:   δ[i] = max(D[i][j]) for all j  Else:   δ[i] = min(D[i][j]) for all j, where ρ[j] > ρ[i].4:Select cluster centers.Identify points with high ρ and δ as cluster centers.5:Assign cluster labels.  For each non-center data point i:   Assign to the cluster of the nearest point j with ρ[j] > ρ[i].

**Figure 8:**
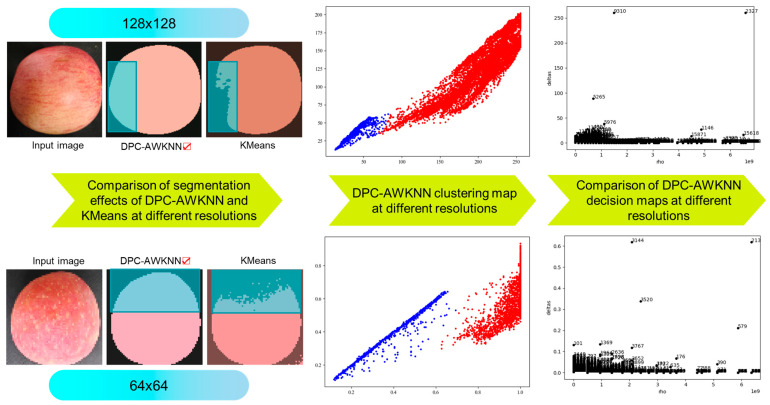
DPC-AWKNN algorithm fruit segmentation results.

To demonstrate the effectiveness of the proposed DPC-AWKNN algorithm in apple segmentation tasks, we evaluated the segmentation performance at different resolutions. We plotted the clustering, density decision graph, and clustering distribution graph and compared the segmentation results with the current mainstream K-Means [24] clustering algorithm. The specific visualization results are shown in Figure 8. In the decision and clustering graphs, it is evident that DPC-AWKNN accurately identifies the optimal decision boundary across different resolutions. In the segmentation comparison, the K-Means clustering algorithm fails to accurately segment areas with weaker illumination, whereas the DPC-AWKNN algorithm precisely segments the entire fruit, showing better performance in low-light edge segmentation. The algorithm exhibits strong anti-interference capabilities, meeting the requirements for fruit segmentation tasks in apple grading pipelines.

#### 2.4.3. Apple Color Feature Extraction

The color features of apples are key indicators in grading tasks. Based on the segmented apple images, this paper sequentially converts the RGB color space of the front and rear views into the HSV color space. Using the threshold range in the HSV color space that represents a color similar to apple red, the red regions are extracted. The HSV range for extracting apple color characteristics is 0–12 or 170–180 for H, 30–255 for S, and 50–200 for V. Subsequently, the red coloring ratio of the apple is calculated, with the specific calculation process shown in Equation (9).

(9) $$ratio=\frac{pixel_{red}}{pixel_{all}}$$

In this context, $pixel_{red}$ represents the number of red pixels in the apple, while $pixel_{all}$ denotes the total number of pixels in the apple.

#### 2.4.4. Apple Diameter Feature Extraction

The apple diameter feature refers to the maximum diameter of the cross-section perpendicular to the axis, extending from the stem to the calyx. This feature is a crucial indicator for apple grading. For Red Fuji apples, a diameter of 60 mm or more qualifies them as grade apples, otherwise they are considered substandard. Due to the irregular shape of apples, accurately calculating the maximum cross-sectional diameter and extracting diameter feature information remains a challenge. Most current studies use other geometric parameters, such as the minimum enclosing circle of the apple contour, to extract feature information. This paper proposes a method that combines the minimum enclosing rectangle and the minimum enclosing circle to extract diameter features. The diameter features are extracted based on a top view. First, the minimum enclosing rectangle [25] is calculated. To enhance the accuracy of diameter measurement, the minimum enclosing circle [26] is introduced as an auxiliary measure. The results obtained from these two methods are averaged to determine the pixel diameter of the apple image. Using a ping-pong ball (with an actual diameter of 40 mm) as a reference, the actual apple diameter is indirectly calculated.

#### 2.4.5. Apple Shape Feature Extraction

In current apple grading technologies, the research on shape features is limited. However, high-quality apples also possess a good shape. Therefore, this study introduces the concept of shape features to further enhance apple grading quality. Apples are generally spherical, and their grading is determined by their similarity to a standard circular shape, known as the shape index. The Canny [27] algorithm is a widely used method for edge detection tasks. It begins by applying a Gaussian filter to the image to suppress noise, followed by the computation of horizontal and vertical gradients using the Sobel operator. The algorithm then examines pixels along the gradient direction, suppressing non-maximum values to retain only local maxima. This process helps distinguish between strong and weak edges, ultimately connecting them to form continuous edges. This paper utilizes the Canny algorithm in conjunction with the minimum enclosing rectangle to calculate the apple shape index. The segmented RGB image is converted to a grayscale image, and the Canny algorithm is employed to detect the edges of the fruit body. The edge image of the apple is used to calculate the vertical and horizontal diameters via the minimum enclosing rectangle, leading to the determination of the apple shape index. The specific calculation process is detailed in Equation (10).

(10) $$E=\frac{h}{w}$$

In this context, h represents the vertical diameter, while w represents the horizontal diameter.

**Figure 9:**
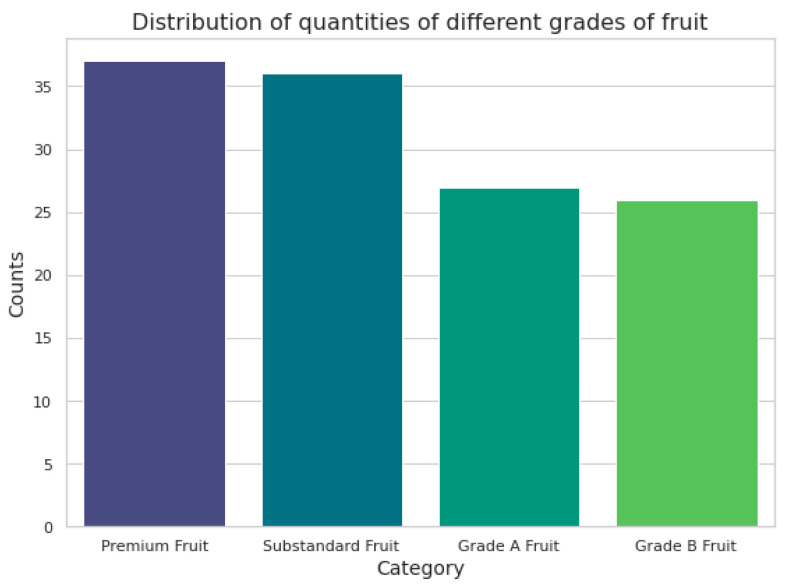
Distribution of apple quantities across different grades.

Based on the aforementioned image preprocessing strategies and feature extraction methods, the MB Filter was initially employed to suppress the noise in apple images. Adaptive histogram equalization was then applied in the L color space to correct areas with uneven surface brightness. The DPC-AWKNN method was utilized to accurately segment the apple body. Subsequently, specific feature extraction techniques were used to derive the color, diameter, and shape features of 126 apples. These apples were then classified into corresponding grades according to apple grading standards. The dataset was split into training and testing sets in a 7:3 ratio. The distribution of apples across the four grades is depicted in Figure 9.

### 2.5. Apple Grading Model

The integration of machine learning classification designs into smart agriculture is a promising area with several representative cases. Mainstream classification models include linear models, K-Nearest Neighbors (KNN) [28], Support Vector Machines (SVMs) [29], the Multi-Layer Perceptron (MLP) [30], and ensemble learning methods, such as Random Forests (RFs) [31], Bagging Classifiers (BCs) [32], and Gradient Boosted Decision Trees (GBDTs) [33]. Each of these models has unique advantages and limitations. Linear Models: They are simple and easy to interpret, making them widely used. However, they perform poorly on non-linearly separable data. Decision trees offer good interpretability but tend to overfit on low-dimensional data when used alone. Ensemble learning enhances overall model accuracy and stability by combining predictions from multiple models. Random forests and gradient boosting machines can capture complex patterns in data due to their construction of multiple tree models, providing robustness and generalizability. In an operational pipeline, rapidly and accurately determining the grade of apples is crucial. The performance of the apple grading model directly affects the grading accuracy. In this study, image processing techniques were utilized to extract three key features: the color ratio, diameter, and shape of apples. These features were used for grading labels, and a dataset for apple grading was constructed. The GBDT algorithm was selected to develop an efficient apple grading model.

GBDT is an ensemble learning algorithm that uses classification and regression trees (CARTs) as weak learners. For each new learner, the residuals from the current learners are used as labels for training the next learner. By fitting the negative gradient of the current model’s loss function, it incrementally minimizes the loss through multiple rounds of iterative training. The result is a GBDT model with strong generalization capabilities. The input apple feature dataset is described by $D=\left\{({\left(x_{c},x_{d},x_{s}\right)}_{i},{\left(y_{g}\right)}_{i})|i=1,2,\cdots ,N\right\}$, where $x_{c}$ represents the color ratio feature, $x_{d}$ represents the diameter feature, $x_{s}$ represents the shape feature, and $y_{g}$ represents the apple’s grade. Since apple grading is a classification problem, the log-likelihood loss function was chosen to evaluate the model. The specific classification process is shown as Algorithm 2.
**Algorithm 2: GBDT**Input: Apple feature data D. Output: Powerful learner $\hat{f}(x)$.1:Initialize $f_{0}(x)={argmin}_{\rho }\sum_{n=1}^{N}L(y_{n},\rho )$ // Initialize the weak learner.2:For m = 1 to M: 
  (a)For i = 1, 2, 3, …, N compute // Calculate the negative gradient.$r_{mi}=-{\left[\frac{\partial L(y_{i},f(x_{i}))}{\partial f(x_{i})}\right]}_{f(x)=f_{m-1}(x)},i=1,\cdots ,N$  (b)The mth CART regression tree is constructed based on $r_{mi}$ and ${\left(x_{c},x_{d},x_{s}\right)}_{i}$ and the regions corresponding to its leaf nodes.$R_{mj},j=1,2,\cdots ,J$  (c)For j = 1, 2, 3, …, J_m_ compute // Calculate its best fit for leaf node regions.$\rho_{mj}=argmin\sum_{x_{i}\in R_{mj}}L(y_{i},f_{m-1}(x_{i})+\rho )$  (d)Update $f_{m}(x)=f_{m-1}(x)+\sum_{j=1}^{J}\rho_{mj}I(x\in R_{mj})$ // Progressively stronger weak learners.3:Output $\hat{f}(x)=f_{M}(x)=f_{0}(x)+\sum_{m=1}^{M}\sum_{j=1}^{J}\rho_{jm}I(x\in R_{jm})$ // After M rounds of iterative training until the model converges, the strong learner is obtained.

## 3. Experiment and Result Analysis

### 3.1. Experimental Environment and Parameter Settings

The experiments in this study were based on the CentOS 7.9.2009 operating system. The processor used was a 12 vCPU Intel(R) Xeon(R) Platinum 8255C CPU@2.50GHz. Considering different model sizes and their varying memory requirements, an NVIDIA GeForce RTX 3090 graphics card with 24 GB of memory was selected. The integrated development environment was Miniconda3, and the development language was Python (version 3.8.0). The fruit stem detection model was built using two mainstream deep learning frameworks: PyTorch (version 1.11.0) and MMDetection (version 3.2.0). CUDA version 11.3 was used to accelerate the model training process. The input image resolution was 640 × 640. The Adam optimizer, which combines momentum optimization with adaptive learning rate strategies, was used to optimize the model training process, with an initial learning rate of 1 × 10^−3^. Each batch of images passed into network training consisted of 64 images. Both the baseline model and the improved model were trained for 200 epochs according to these parameter settings, with mosaic data augmentation disabled for the last 10 epochs. The apple grading model was constructed using the scikit-learn machine learning library (version 1.3.2).

### 3.2. Fruit Stem Detection Experiments

#### 3.2.1. Fruit Stem Detection Model Evaluation Metrics

This study used common evaluation metrics for detection tasks to assess the detection performance of the fruit stem detection model, including Precision (P), Recall (R), mean Average Precision (mAP), Average Precision (AP), model computational cost (GFLOPs), and FPS (frames per second). The specific calculations are shown in Equations (11)–(14).

(11) $$P=\frac{TP}{TP+FP}$$

(12) $$R=\frac{TP}{TP+FN}$$

(13) $$AP=\int_{0}^{1}P(r)dr$$

(14) $$mAP=\frac{1}{n}\sum_{i=0}^{n}AP_{i}$$

where n refers to the number of categories, and in the reported experiment there was only one category: fruit stem. mAP refers to the average precision calculated for the fruit stem category, and mAP@0.5 represents the detection performance of the model when the IoU (Intersection over Union) threshold is set to 0.5. In contrast, mAP@0.5:0.95 is the average mAP calculated across multiple IoU thresholds ranging from 0.5 to 0.95 with a step size of 0.05, providing a comprehensive evaluation of the model’s performance under different IoU thresholds. P indicates the proportion of instances detected as fruit stems that are actually fruit stems, measuring the model’s false detection rate. R indicates the proportion of actual fruit stem instances that were correctly detected, measuring the model’s misdetection rate. TP represents the number of fruit stems that the model correctly predicted, FP represents the number of background instances the model incorrectly detected as fruit stems, and FN represents the actual number of fruit stems that the model failed to detect.

Furthermore, FPS and GFLOPs were introduced as metrics for evaluating the inference performance of the models. FPS denotes the number of image frames a model can process per second. In the field of object detection, a model capable of running at frame rates of 30 FPS or above is considered to possess real-time detection capabilities. A higher FPS indicates that the model can be deployed on hardware with lower computational power, thus enhancing its applicability in environments with limited computing resources. GFLOPs, on the other hand, is a metric that measures the computational complexity of an algorithm, typically related to the model’s number of parameters and operations.

#### 3.2.2. Feature Extraction Network Comparison Experiment

To demonstrate the effectiveness of the FasterNet-t0 feature extraction network, we designed comparative experiments involving various feature extraction networks. These included mainstream lightweight networks, such as MobileNetv3 [34] and GhostNet [35], and three different sizes of the FasterNet-t series. To ensure the fairness of the comparisons, the feature fusion network PANet from YOLOv5s was used consistently. The specific results of the comparative experiments on fruit stem detection using different feature extraction networks are presented in Table 2.

Based on the experimental results in Table 2, it can be concluded that for the task of fruit stem detection, the FasterNet-t0 network demonstrates optimal overall performance. The model size was reduced by 2.8 MB, GFLOPs decreased by 4.6, and the accuracy remained nearly unchanged. This indicates that the PConv operator reduces redundant computations and lowers the model’s complexity to a certain extent. Although the MobileNetv3 and GhostNet networks significantly reduced the number of parameters and computational cost, their mAP@0.5:0.95 values suffered substantial losses, making them unsuitable for high-accuracy requirements in practical fruit stem detection tasks. Additionally, while FasterNet-t1 and FasterNet-t2 showed varying degrees of accuracy improvement, their computational loads and model sizes were relatively large. Therefore, considering a comprehensive comparison of different models, FasterNet-t0 emerged as the best choice for a feature extraction network.

#### 3.2.3. Ablation Study on the Improvement Process

This paper conducted a series of ablation experiments to demonstrate the effectiveness of the proposed improvement strategies for enhancing fruit stem detection tasks. Based on the YOLOv5s detection network, the study specifically explored the use of FasterNet-t0 to improve the feature extraction network, the introduction of DBB-PANet to enhance the feature fusion network, and the combination of both methods for further improvement. The detailed ablation experiment results for different improvement strategies in the fruit stem detection task are presented in Table 3.

Based on the ablation experiment results in Table 3, it is evident that using FasterNet-t0 as a feature extraction network reduces GFLOPs by 4.6 and decreases the model size by 2.8 MB, achieving model lightweighting. This indicates that the PConv operator in FasterNet effectively reduces memory access frequency, with accuracy remaining almost on par with the baseline model. Utilizing DBB-PANet to improve the feature fusion network boosts the P by 0.3% and R by 2.3%, with a slight increase in accuracy. This suggests that DBB-PANet enhances performance in fruit stem detection tasks, effectively improving the recognition capability for stems of varying sizes through a multi-scale structural re-parameterization strategy. When both strategies are combined to improve the YOLOv5-s model, the precision is increased by 2.7%, the mAP@0.5 value rises by 0.8%, and the GFLOPs is reduced by 1.8 compared to the baseline model. This demonstrates that DBB-PANet fully integrates the feature information extracted by the FasterNet-t0 network, resulting in improved detection accuracy while simultaneously reducing computational demand.

**Figure 10:**
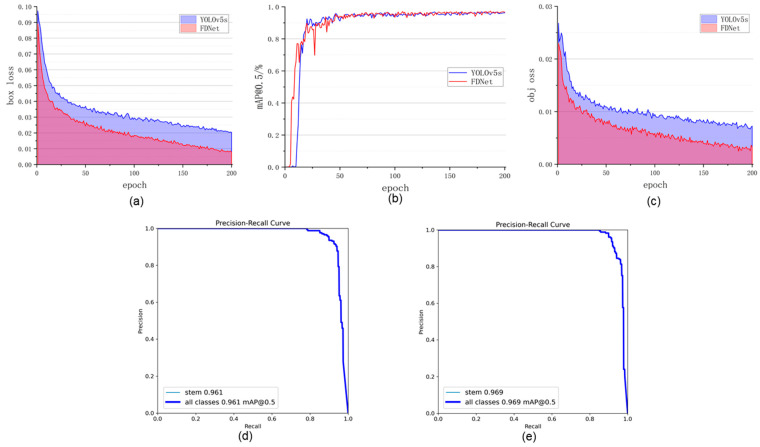
Comparison of different evaluation metrics between FDNet and YOLOv5-s. (**a**) bbox loss training curve. (**b**) mAP@0.5 training curve. (**c**) obj_loss training curve. (**d**) PR Curves of YOLOv5-s on the validation set. (**e**) PR Curves of FDNet on the validation set.

To more intuitively demonstrate that the proposed FDNet outperforms the baseline YOLOv5-s model, we visualized the bbox_loss, obj_loss, and mAP@0.5 during the training process, as shown in Figure 10. From Figure 10a,c, it is apparent that both loss curves for FDNet are superior to those of YOLOv5-s during training, indicating better model convergence. The mAP@0.5 value for FDNet also exceeds that of YOLOv5-s, as depicted in Figure 10b. Additionally, the Precision–Recall Curve was used to further analyze the detection accuracy of FDNet and YOLOv5-s on the validation set. Figure 10d presents the detection results for FDNet, while Figure 10e shows the results for YOLOv5-s. Notably, the mAP@0.5 value for FDNet is 0.969, which is higher than that of YOLOv5-s.

#### 3.2.4. Comparative Experiments on Model Compression

To thoroughly demonstrate the impact of pruning strategies on model performance, this study conducted comprehensive comparative experiments using different model compression strategies. The three mainstream pruning algorithms selected were Slim [36], L1 [37], and Lamp. Additionally, key metrics, such as inference time and FPS, were evaluated on a resource-constrained GPU (1080Ti). The specific comparative results of different pruning strategies are presented in Table 4.

**Figure 11:**
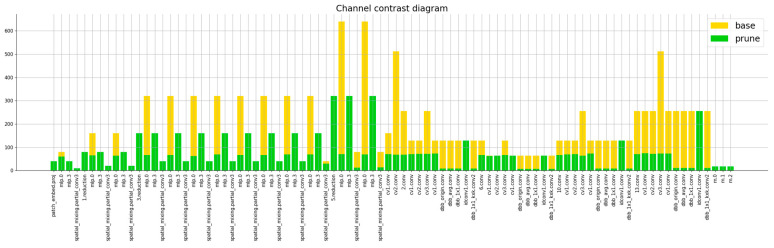
Comparison of the number of channels in the model before and after pruning.

According to the results of the model compression comparative experiments in Table 4, when the compression ratio is set to 2.0, the Lamp pruning algorithm demonstrates the best overall performance across various evaluation metrics. Compared to the Slim and L1 pruning algorithms, the Lamp algorithm results in the smallest model size post-pruning, with the lowest decrease in mAP@0.5:0.95. On low-resource devices, it achieves the fastest inference speed, with a Latency of 0.268 and an FPS of 372.8, highlighting the effectiveness of the Lamp pruning algorithm. This method of adaptive structured pruning at the channel level preserves the original convolutional structure, making it more hardware-friendly. Additionally, to explore the optimal model compression strategy, further comparative experiments with different compression ratios were conducted using the Lamp pruning algorithm. It was found that at a compression ratio of 4.0, the model exhibited the best compression effects: an mAP@0.5 value of 96.6, a GFLOPs of 3.4, a model size of 2.5, a Latency of 0.200, and an FPS of 499.5. To visually illustrate the pruning effects, the changes in the number of channels in each layer of the model before and after using Lamp pruning with a compression ratio of 4.0 were plotted, as shown in Figure 11.

#### 3.2.5. Model Comparative Experiments

By conducting a series of comparative experiments, this study demonstrated the superiority of the proposed FDNet and FDNet-p models in the task of stem detection. These models were thoroughly compared with state-of-the-art mainstream object detection models across various evaluation metrics. As FDNet is characterized as a lightweight single-stage object detection algorithm, the comparison primarily focused on lightweight single-stage detection algorithms from the YOLO series. These included YOLOv3 [38], YOLOv5 [39], YOLOv6 [40], YOLOv8 [41], YOLOv9 [42], YOLOv10 [43], and YOLOv11 [44]. Additionally, to fully validate the performance of FDNet, the comparison included mainstream non-YOLO-series object detection algorithms, such as Faster R-CNN [45], GFL [46], RTMDet [47], YOLOx [48], and TOOD [49]. The specific comparative experiment results are detailed in Table 5.

Based on the quantitative analysis results in Table 5, it is evident that the proposed FDNet (uncompressed) model for stem detection exhibits superior performance compared to other detection models, achieving the highest mAP@0.5 value of 96.9%. Within the YOLO series of object detection algorithms, YOLOv6s shows the best detection performance; however, it has significant parametric and computational requirements, necessitating higher resource availability. In comparison, the FDNet algorithm has a higher mAP@0.5 by 0.09 and a nearly equivalent mAP@0.5:0.95 value, yet it features a significantly lower GFLOPs and model size than YOLOv6s. When compared to models with similar computational demands, the FDNet model achieves higher detection accuracy. In the realm of non-YOLO object detection algorithms, FDNet remains superior in terms of detection accuracy, computational load, and model size. After applying compression strategies, the FDNet-p model achieved a Precision (P) value of 96.3%, a Recall (R) value of 89.4%, an mAP@0.5 of 96.6%, and an mAP@0.5:0.95 of 67.9%, with only a 3.4 GFLOPs and a model size of just 2.5 MB. These metrics indicate that FDNet-p achieves a near-lossless compression, highlighting the efficiency of the Lamp pruning algorithm. Among many object detection models, the FDNet-p model has the lowest resource consumption. After applying LAMP pruning, the computational needs are drastically reduced, with the GFLOPs value at just 3.4, while maintaining high accuracy.

**Figure 12:**
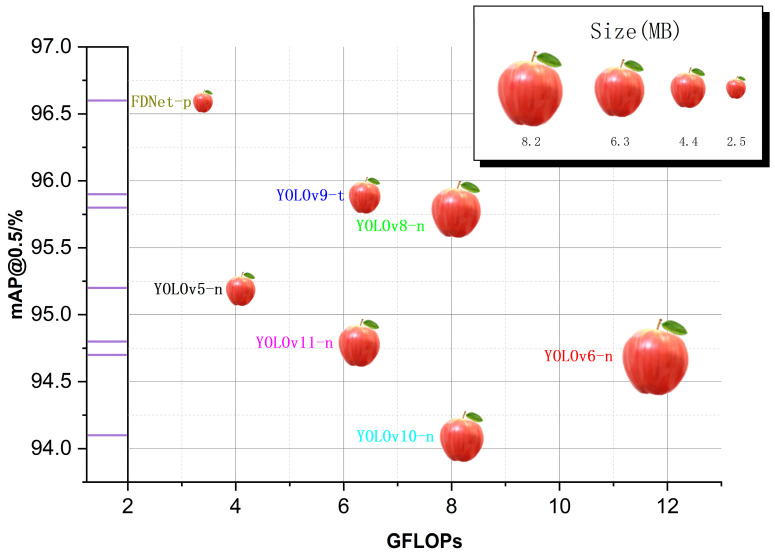
Performance comparison of lightweight detection models.

To visually demonstrate the superior performance of the compressed FDNet model (FDNet-p), a comparative analysis with lightweight YOLO-series models (v5, v6, v8, v9, v10, and v11) was conducted. The visual results are shown in Figure 12. In the stem detection task, FDNet-p requires the lowest GFLOPs compared to other lightweight models, has the smallest model size, and is easily deployable on resource-constrained devices on production lines, all while maintaining the highest mAP@0.5 value to meet the precision requirements of stem detection tasks.

**Figure 13:**
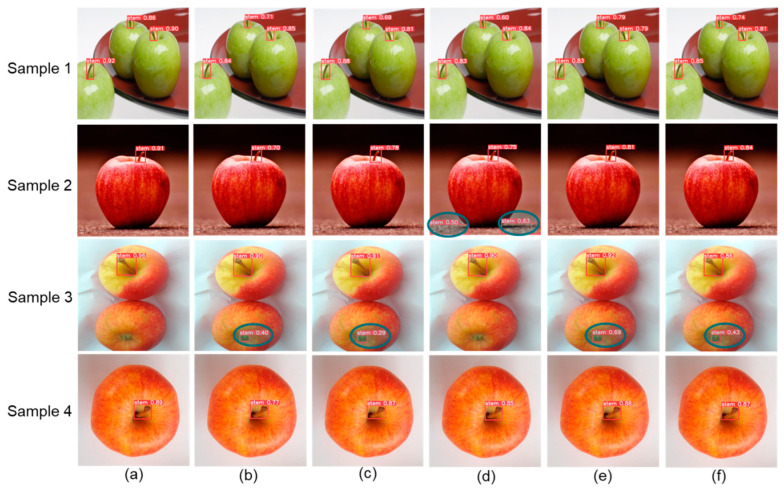
Comparative effectiveness of different detection algorithms. (**a**) FDNet-p. (**b**) YOLOv11-n. (**c**) YOLOv10-n. (**d**) YOLOv9-n. (**e**) YOLOv8-n. (**f**) YOLOv6-n.

By presenting detection effect images from real-world scenarios, this study further demonstrates the strong robustness and high detection accuracy of the proposed FDNet-p. For fairness in evaluating detection performance, lightweight detection models from the YOLO series (v5, v6, v8, v9, v10, and v11) were also used for comparison. The specific detection result comparisons are illustrated in Figure 13.

Four different scenarios were selected for analysis: (I) Multi-target with complex background (Sample 1): FDNet-p achieved the highest detection accuracy across the three different apples, while other models demonstrated lower accuracy. (II) Single target with complex background (Sample 2): The YOLOv9-n model mistakenly identified the background as a stem. (III) Simple background with sepal interference (Sample 3): Other models misidentified sepals as stems to varying degrees, while FDNet-p accurately identified the stems. (IV) Overhead view angle (Sample 4): FDNet-p exhibited the best detection performance.

### 3.3. Apple Grading Experiments

#### 3.3.1. Apple Grading Model Evaluation Metrics

In the study of the apple grading model, we used a variety of evaluation metrics to comprehensively analyze the model’s performance. These metrics included Precision (P), Recall (R), Jaccard Score, and F1 Score. The F1 Score and Jaccard Score were used to measure the overall performance and accuracy of the model across different categories. To provide a more comprehensive analysis of the model’s performance and a better understanding of its effectiveness in multi-class problems, we introduced three different averaging methods: Micro, Macro, and Weighted. The Micro Average evaluated overall performance, reflecting the proportional impact of each sample; the Macro Average emphasized the importance of each category equally; and the Weighted Average addressed class imbalance issues by accounting for the size of each class. These metrics reflected the model’s sensitivity, correctness, and applicability from different perspectives. The specific calculation formulas are shown in Table 6, where C represents the number of categories, *N* represents the total number of samples, and *n_i_* represents the number of samples in the i-th category.

#### 3.3.2. Comparative Analysis of Apple Grading Experiments

The study on apple grading algorithms in this paper was based on three features: color, diameter, and shape. By setting up a series of comparative experiments with mainstream machine learning algorithms, multiple evaluation metrics were used to assess the performance of different algorithms in apple grading. To determine the best apple grading model, six apple grading models were established, and their performance was evaluated using 10-fold cross-validation to effectively reduce bias in the model evaluation process. The ensemble learning algorithm models included BC, RF, and GBDT, while the non-ensemble learning algorithms included KNN, SVM, and MLP. The specific experimental results of the different apple grading algorithms are shown in Table 7.

Based on the cross-validation experimental results in Table 7, it can be observed that the overall performance of non-ensemble learning algorithms is weaker than that of ensemble learning algorithms. Among the ensemble learning algorithms, the GBDT apple grading algorithm demonstrated the best overall performance across various evaluation metrics. Specifically, compared to other ensemble learning algorithms, the GBDT algorithm achieved the best F1 Score values under three different averaging methods, which were 0.9513, 0.9476, and 0.9506, respectively. It also attained the highest values for Jaccard Score, P, and R, highlighting the GBDT algorithm’s excellent classification ability, robust performance, and strong generalization capability, making it suitable as an apple grading model.

**Figure 14:**
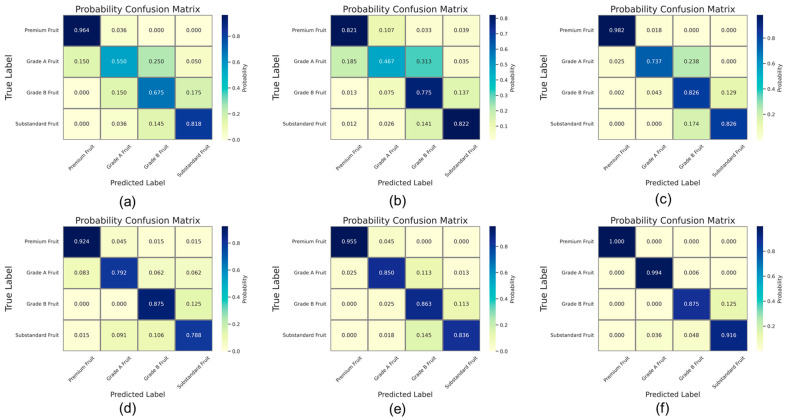
Visualization of apple grading model performance comparison. (**a**) KNN Model. (**b**) SVM Model. (**c**) MLP Model. (**d**) RF Model. (**e**) BC Model. (**f**) GBDT Model.

To more effectively verify the superior performance of the GBDT apple grading algorithm, we visualized the prediction probabilities of different algorithms on the test set using confusion matrices. This visualization illustrates the models’ prediction accuracy across various categories and allows for further analysis of the grading performance of different models on four apple grades, as detailed in Figure 14. Overall, it is apparent that among non-ensemble algorithms, the MLP algorithm achieves relatively better grading performance. In contrast, the KNN and SVM algorithms struggle to effectively distinguish between Grade A and Grade B apples, with the KNN algorithm having a prediction probability of only 0.550 for Grade A Fruit and a 0.25 probability of misclassifying it as Grade B Fruit. Among ensemble learning algorithms, RF, BC, and GBDT all achieve relatively accurate grading for four apple grades. The GBDT algorithm demonstrates the best grading performance, effectively capturing the distinguishing features among different apple grades, with prediction probabilities of 1.000, 0.994, 0.875, and 0.916 for the four apple grades, respectively, thereby achieving a near-perfect classification.

## 4. Discussion

In apple automatic grading systems, research on single traits such as color, fruit diameter, and fruit shape primarily focuses on image processing techniques. Although stem detection is less common, there are reports of its realization through image processing methods. In the study of apple feature extraction, multi-feature fusion, and appearance grading technology based on the four aforementioned indicators, this paper is the first to apply stem detection based on deep learning and fruit segmentation using cluster algorithms, as well as fruit color, fruit diameter, and fruit shape extraction through image processing. Moreover, it integrates these with feature fusion technology based on ensemble learning methods for apple automatic grading. We introduced the stem characteristic into apple grading for the first time and designed a lightweight stem detection algorithm, FDNet-p, which, among various detection models, satisfies actual production requirements both in terms of computational load and detection accuracy, providing a new design approach for detection tasks. Subsequently, we proposed an improved DPC-AWKNN method to enhance fruit segmentation effects, followed by the extraction of three feature information types—color, fruit diameter, and fruit shape. Finally, through extensive comparative experiments, the GBDT algorithm was identified as more reliable for feature fusion and the execution of the apple grading task.

**Figure 15:**
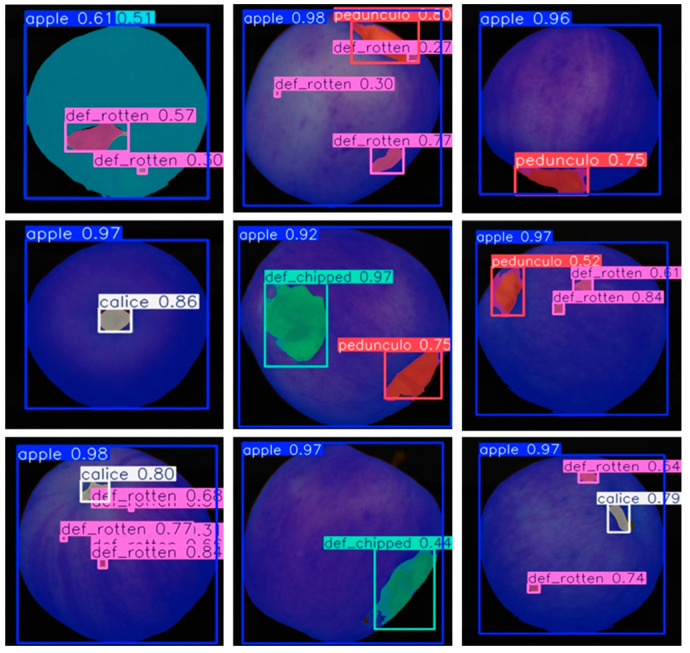
Segmentation of apple defect areas.

However, this study acknowledges certain limitations and shortcomings in practical applications, necessitating further improvements and innovations. Primarily, the current apple grading task does not account for surface defects, which are crucial criteria in commercial grading. In commercial standards, Premium Fruit require nearly flawless surfaces, while Grade A Fruit and Grade B Fruit may permit minor surface defects. Therefore, to align apple grading with commercial standards, the extraction of surface defects is an essential issue that must be addressed in future research. Our team is currently exploring deep learning algorithms to analyze surface defect characteristics of apples and has already achieved some preliminary results, as illustrated in Figure 15. In addition, within the context of the apple grading pipeline, our current research primarily focuses on the segmentation of individual apples. For multi-object apple segmentation, we plan to explore the use of deep learning-based segmentation algorithms in the future to achieve the segmentation of both single and multiple apples.

Moreover, the current study focused on feature extraction and subsequent apple grading model experiments based on a small sample size of apples. These apples had already undergone a simple manual grading process before reaching the market, which limited the range of feature information they expressed and consequently restricted the generalizability of the grading model. Therefore, it is crucial to construct a large-scale apple sample dataset, particularly one comprising freshly harvested apples during their peak season, to ensure that the distribution of sample features closely resembles the real conditions found in orchards. In the future, we plan to harvest large quantities of apple samples from orchards to develop a comprehensive and diverse dataset.

Lastly, this study does not address internal quality grading technologies, such as those assessing apple sweetness, firmness, and watercore disease. Future work will focus on developing grading technologies for internal apple qualities, integrating them with the current study’s external quality grading methods to create a non-destructive and multi-criteria apple grading system integrating internal and external quality indicators.

## 5. Conclusions

The study presents a novel architecture for the apple grading task, which, for the first time, organically integrates computer vision, image processing, and machine learning technologies. This innovative approach involves an advanced automated apple grading technology. The apple grading system introduces the stem index for the first time and proposes a high-precision lightweight stem detection algorithm, FDNet-p. It employs the FasterNet network for low-memory-access feature extraction of the stem and includes a DBB-PANet network to integrate stem feature information, thereby enabling rapid stem detection. This allows for the screening out of stemless apples and introduces an auxiliary positioning concept to prevent premature or delayed camera capture. For the apple feature extraction process, an improved DPC-AWKNN segmentation algorithm is proposed, along with an adaptive brightness equalization strategy to enhance fruit segmentation. Image processing techniques are employed to extract multiple apple features: color features are extracted via color space transformation and threshold segmentation, while diameter and shape features are extracted using Canny edge detection, minimum enclosing rectangle, and minimum enclosing circle techniques, thus mapping to real-world manual grading scenarios. An intelligent multi-feature grading model for apples was developed based on the GBDT algorithm. Experimental results indicate that in the stem detection task, the proposed lightweight FDNet-p model outperformed numerous mainstream detection models, achieving the best detection effect with an mAP@0.5 value of 96.6%. It has the lowest computational load, with a GFLOPs as low as 3.4 and an FPS value of 499.5, enabling rapid and accurate stem detection. In the apple grading experiments, compared to various classification models, the GBDT grading model demonstrated optimal comprehensive performance, with weighted Jacard Score, Precision, Recall, and F1 Score values of 0.9506, 0.9196, 0.9683, and 0.9513, respectively.

In the future, we will focus on further innovative designs of stem detection models to enhance the accuracy of stem detection in apple grading tasks. By employing segmentation-based computer vision methods, we will extract defect feature information from apples and advance research on internal grading techniques, enabling the extraction of richer and more comprehensive apple feature data. This work will integrate non-destructive internal and external apple grading technologies, culminating in the development of a complete non-destructive inspection and grading system for apples. This system will be deployed in apple grading production lines in agriculture, aiming to improve product quality, deliver higher economic returns to apple growers, and further enhance food safety standards.

## Figures and Tables

**Table 1:** Apple grading customized standards.

Attribute	Apple Grade			
	Premium Fruit	Grade A Fruit	Grade B Fruit	Substandard Fruit
Stem Feature ↓	Intact	Intact	Intact (minor damage allowed)	Does not meet the requirements for the corresponding grade
Color Feature ↓	≥90%	≥75%	≥60%	
Diameter Feature ↓	≥80 mm	≥70 mm	≥65 mm	
Shape Feature ↓	≥0.8	≥0.7	≥0.6	

↓ represents the order of feature priority.

**Table 2:** Comparative experiments on different feature extraction networks.

Backbone	P/%	R/%	mAP@0.5/%	mAP@0.5:0.95/%	GFLOPs	Size/MB
MobileNetv3	93.0	90.5	95.3	67.0	6.5	8.7
GhostNet	89.1	93.7	95.2	65.7	5.9	6.7
FasterNet-t0	95.1	91.5	95.8	69.5	11.2	11.0
FasterNet-t1	95.2	91.0	96.1	70.4	20.0	18.1
FasterNet-t2	93.7	92.1	96.9	69.7	37.9	32.5

**Table 3:** Ablation experiments on the improvement process.

FasterNet-t0	DBB-PANet	P/%	R/%	mAP@0.5/%	mAP@0.5:0.95/%	GFLOPs	Size/MB
		93.0	91.9	96.1	70.1	15.8	13.8
√		95.1	91.5	95.8	69.5	11.2	11.0
	√	93.3	94.2	96.2	70.2	18.1	16.6
√	√	95.7	91.0	96.9	69.8	13.6	14.2

√ represents the selected improvement strategy.

**Table 4:** Comparative experiments on model compression.

Method	Compression Ratio	P/%	R/%	mAP@ 0.5/%	mAP@ 0.5:0.95/%	GFLOPs	Size/MB	Latency/ms	FPS (Batch = 32)
FDNet	0.0	95.7	91.0	96.9	69.8	13.6	14.2	0.448 ± 0.169	228.1
+lim	2.0	95.9	91.5	96.6	68.2	6.8	6.4	0.277 ± 0.072	360.4
+1	2.0	95.4	93.1	96.1	68.2	6.8	5.5	0.275 ± 0.161	363.0
+AMP	2.0	95.9	89.9	96.1	68.7	6.8	5.3	0.268 ± 0.032	372.8
+AMP	3.0	93.1	93.1	96.2	67.2	4.5	3.3	0.240 ± 0.144	416.1
+AMP	4.0	96.3	89.4	96.6	67.9	3.4	2.5	0.200 ± 0.113	499.5

**Table 5:** Comparative experiments on different detection models.

Model Name	P/%	R/%	mAP@0.5/%	mAP@0.5:0.95/%	GFLOPs	Size/MB
YOLO Series						
YOLOv3-t	94.9	89.2	95.6	70.0	18.9	24.4
YOLOv5-n	95.9	89.9	95.2	68.6	4.1	3.7
YOLOv5-s	93.0	91.9	96.1	70.1	15.8	13.8
YOLOv6-n	95.0	91.0	94.7	69.8	11.8	8.3
YOLOv6-s	96.1	92.2	96.0	70.4	44	32.8
YOLOv8-n	92.1	87.3	95.8	68.9	8.1	6.2
YOLOv9-t	95.1	89.9	95.9	68.3	6.4	4.0
YOLOv10-n	92.2	86.2	94.1	65.1	8.2	5.5
YOLOv11-n	95.9	91.0	94.8	67.4	6.3	5.2
Non-YOLO Series						
FasterRCNN	96.0	90.2	96.0	69.9	134	315.1
GFL	95.6	89.2	96.6	68.2	128	245.8
RTMDet	95.4	88.8	95.3	68.3	8.1	79.5
YOLOx	95.9	88.2	95.5	67.4	7.6	61.5
TOOD	94.8	91.4	96.2	67.6	123	243.9
Ours (FDNet)	95.6	91.0	96.9	69.8	13.6	14.2
Ours (FDNet-p)	96.3	89.4	96.6	67.9	3.4	2.5

**Table 6:** Evaluation metrics for the grading model.

Items	Categories	Equation
Jaccard Score	Micro	$\frac{\sum_{i=1}^{N}\left|y_{i}\cap {\hat{y}}_{i}\right|}{\sum_{i=1}^{N}\left|y_{i}\cup {\hat{y}}_{i}\right|}$
	Macro	$\frac{1}{C}\sum_{i=1}^{C}\left|\frac{y_{i}\cap {\hat{y}}_{i}}{y_{i}\cup {\hat{y}}_{i}}\right|$
	Weighted	$\sum_{i=1}^{C}\frac{n_{i}}{N}\times \left|\frac{y_{i}\cap {\hat{y}}_{i}}{y_{i}\cup {\hat{y}}_{i}}\right|$
P	Micro	$\frac{\sum_{i=1}^{C}{TP}_{i}}{\sum_{i=1}^{C}\left({TP}_{i}+{FP}_{i}\right)}$
	Macro	$\frac{1}{C}\sum_{i=1}^{C}\frac{{TP}_{i}}{{TP}_{i}+{FP}_{i}}$
	Weighted	$\sum_{i=1}^{C}\frac{n_{i}}{N}\times \frac{{TP}_{i}}{{TP}_{i}+{FP}_{i}}$
R	Micro	$\frac{\sum_{i=1}^{C}{TP}_{i}}{\sum_{i=1}^{C}\left({TP}_{i}+{FN}_{i}\right)}$
	Macro	$\frac{1}{C}\sum_{i=1}^{C}\frac{{TP}_{i}}{{TP}_{i}+{FN}_{i}}$
	Weighted	$\sum_{i=1}^{C}\frac{n_{i}}{N}\times \frac{{TP}_{i}}{{TP}_{i}+{FN}_{i}}$
F1 Score	Micro	$\frac{2\times P(micro)\times R(micro)}{P(micro)+R(micro)}$
	Macro	$\frac{1}{C}\sum_{i=1}^{C}F1\;{Score}_{i}$
	Weighted	$\sum_{i=1}^{C}\frac{n_{i}}{N}F1\;{Score}_{i}$

**Table 7:** Comparison of 10-fold cross-validation results for different apple grading algorithms.

	F1 Score			Jaccard Score			P			R		
Model	Micro	Macro	Weighted	Micro	Macro	Weighted	Micro	Macro	Weighted	Micro	Macro	Weighted
KNN	0.8340 ± 0.1143	0.8129 ± 0.1321	0.8263 ± 0.1186	0.7319 ± 0.1711	0.7292 ± 0.1766	0.7465 ± 0.1598	0.8340 ± 0.1143	0.8479 ± 0.1155	0.8733 ± 0.0946	0.8340 ± 0.1143	0.8333 ± 0.1204	0.8340 ± 0.1143
SVM	0.8494 ± 0.0740	0.8199 ± 0.0861	0.8370 ± 0.0808	0.7450 ± 0.1070	0.7271 ± 0.1124	0.7520 ± 0.1071	0.8494 ± 0.0740	0.8792 ± 0.0619	0.8930 ± 0.0599	0.8494 ± 0.0740	0.8375 ± 0.0756	0.8494 ± 0.0740
MLP	0.8968 ± 0.0606	0.8529 ± 0.0968	0.8742 ± 0.0809	0.8068 ± 0.1144	0.7979 ± 0.1153	0.8317 ± 0.0950	0.8968 ± 0.0606	0.9233 ± 0.0495	0.9395 ± 0.0417	0.8962 ± 0.0802	0.8854 ± 0.0723	0.8801 ± 0.0743
RF	0.9128 ± 0.0839	0.9101 ± 0.0844	0.9265 ± 0.0785	0.8651 ± 0.1497	0.8542 ± 0.1269	0.8732 ± 0.1062	0.9199 ± 0.0732	0.9479 ± 0.0490	0.9579 ± 0.0373	0.9288 ± 0.0742	0.9417 ± 0.0573	0.9365 ± 0.0599
BC	0.9353 ± 0.0800	0.9231 ± 0.0806	0.9410 ± 0.0802	0.8619 ± 0.1354	0.8938 ± 0.1193	0.8966 ± 0.1111	0.9353 ± 0.0723	0.9542 ± 0.0512	0.9671 ± 0.0352	0.9436 ± 0.0820	0.9417 ± 0.0573	0.9519 ± 0.0642
GBDT	0.9513 ± 0.0546	0.9476 ± 0.0569	0.9506 ± 0.0549	0.9121 ± 0.0962	0.9146 ± 0.0901	0.9196 ± 0.0864	0.9513 ± 0.0546	0.9604 ± 0.0432	0.9683 ± 0.0325	0.9513 ± 0.0546	0.9542 ± 0.0473	0.9513 ± 0.0546

## Data Availability

The original contributions presented in the study are included in the article, further inquiries can be directed to the corresponding author.

## References

[1] Yu J., Zhang Z., Li Y., Hua W., Wei X., Igathinathane C., Mhamed M., Zhang W., Jiao X., Yang L. (2024). In-field grading and sorting technology of apples: A state-of-the-art review. Comput. Electron. Agric..

[2] Ji W., Wang J., Xu B., Zhang T. (2023). Apple Grading Based on Multi-Dimensional View Processing and Deep Learning. Foods.

[3] Toylan H., Kuscu H. (2014). A Real-Time Apple Grading System Using Multicolor Space. Sci. World J..

[4] Sofu M., Er O., Kayacan M., Cetişli B. (2016). Design of an automatic apple sorting system using machine vision. Comput. Electron. Agric..

[5] Zhao D., Ai Y. (2022). Research on apple size detection method based on computer vision. J. Agric. Mech. Res.

[6] Huang Z., Zhu Q. (2016). Detection of Red Region of Fuji Apple Based on RGB Color Model. Laser Optoelectron. Prog..

[7] Tan Y., Gu B., Ji C., Tian G., Jin L., Li J. (2016). Design of on-line apple grading system based on color and weight. Comput. Eng. Appl..

[8] Qiu G., Peng G., Tao D., Wang Z. (2017). Detection on surface defect of apples by DT-SVM method. Food Mach..

[9] Yu Y., Velastin S.A., Yin F. (2020). Automatic grading of apples based on multi-features and weighted K-means clustering algorithm. Inf. Process. Agric..

[10] Fan Z., Liu Q., Chai J., Yang X., Li H. (2020). Apple detection and grading based on color and fruit-diameter. Comput. Eng. Sci..

[11] Shi X., Chai X., Yang C., Xia X., Sun T. (2022). Vision-based apple quality grading with multi-view spatial network. Comput. Electron. Agric..

[12] Fan S., Liang X., Huang W., Zhang V.J., Pang Q., He X., Li L., Zhang C. (2022). Real-time defects detection for apple sorting using NIR cameras with pruning-based YOLOV4 network. Comput. Electron. Agric..

[13] (2008). Fresh Apple.

[14] Li H., Gu Z., He D., Wang X., Huang J., Mo Y., Li P., Huang Z., Wu F. (2024). A lightweight improved YOLOv5s model and its deployment for detecting pitaya fruits in daytime and nighttime light-supplement environments. Comput. Electron. Agric..

[15] Chen J., Kao S., He H., Zhuo W., Wen S., Lee C.-H., Chan S.-H.G. Run, don’t walk: Chasing higher FLOPS for faster neural networks. Proceedings of the Proceedings of the IEEE/CVF Conference on Computer Vision and Pattern Recognition.

[16] Han B., Lu Z., Zhang J., Almodfer R., Wang Z., Sun W., Dong L. (2024). Rep-ViG-Apple: A CNN-GCN Hybrid Model for Apple Detection in Complex Orchard Environments. Agronomy.

[17] Ding X., Zhang X., Han J., Ding G. Diverse branch block: Building a convolution as an inception-like unit. Proceedings of the IEEE/CVF Conference on Computer Vision and Pattern Recognition.

[18] Ding X., Zhang X., Ma N., Han J., Ding G., Sun J. Repvgg: Making vgg-style convnets great again. Proceedings of the IEEE/CVF conference on computer vision and pattern recognition.

[19] Storath M., Weinmann A. (2018). Fast median filtering for phase or orientation data. IEEE Trans. Pattern Anal. Mach. Intell..

[20] Gavaskar R.G., Chaudhury K.N. (2018). Fast adaptive bilateral filtering. IEEE Trans. Image Process..

[21] Yuan Q., Dai S. (2024). Adaptive histogram equalization with visual perception consistency. Inf. Sci..

[22] Wang Y., Qian J., Hassan M., Zhang X., Zhang T., Yang C., Zhou X., Jia F. (2024). Density peak clustering algorithms: A review on the decade 2014–2023. Expert Syst. Appl..

[23] Putra A. (2023). Fingerprint identification for attendance using euclidean distance and manhattan distance. Sinkron.

[24] Ahmed M., Seraj R., Islam S. (2020). The k-means algorithm: A comprehensive survey and performance evaluation. Electronics.

[25] Chan T., Har-Peled S. (2021). Smallest k-enclosing rectangle revisited. Discret. Comput. Geom..

[26] Liu W., Fu X., Deng Z., Xu L., Jiao J. Smallest enclosing circle-based fingerprint clustering and modified-WKNN matching algorithm for indoor positioning. Proceedings of the 2016 International Conference on Indoor Positioning and Indoor Navigation (IPIN).

[27] Feng Y., Han B., Wang X., Shen J., Guan X., Ding H. (2024). Self-Supervised Transformers for Unsupervised SAR Complex Interference Detection Using Canny Edge Detector. Remote Sens..

[28] Huang A., Xu R., Chen Y., Guo M. (2023). Research on multi-label user classification of social media based on ML-KNN algorithm. Technol. Forecast. Soc. Change.

[29] Wang H., Li G., Wang Z. (2023). Fast SVM classifier for large-scale classification problems. Inf. Sci..

[30] Sabancı K. (2016). Different apple varieties classification using kNN and MLP algorithms. Int. J. Intell. Syst. Appl. Eng..

[31] Belgiu M., Drăguţ L. (2016). Random forest in remote sensing: A review of applications and future directions. ISPRS J. Photogramm. Remote Sens..

[32] Sandag G.A. (2020). A prediction model of company health using bagging classifier. J. Ilmu Pengetah. Dan Teknol. Komput..

[33] Zhang Z., Jung C. (2020). GBDT-MO: Gradient-boosted decision trees for multiple outputs. IEEE Trans. Neural Netw. Learn. Syst..

[34] Zhang J., Cai Y.-Y., Yang D., Yuan Y., He W.-Y., Wang Y.-J. (2023). MobileNetV3-BLS: A broad learning approach for automatic concrete surface crack detection. Constr. Build. Mater..

[35] Du Z., Xu X., Bai Z., Liu X., Hu Y., Li W., Wang C., Li D. (2023). Feature fusion strategy and improved GhostNet for accurate recognition of fish feeding behavior. Comput. Electron. Agric..

[36] Liu Z., Li J., Shen Z., Huang G., Yan S., Zhang C. Learning efficient convolutional networks through network slimming. Proceedings of the IEEE International Conference on Computer Vision.

[37] Kumar A., Shaikh A.M., Li Y., Bilal H., Yin B. (2021). Pruning filters with L1-norm and capped L1-norm for CNN compression. Appl. Intell..

[38] Farhadi A., Redmon J. (2018). Yolov3: An incremental improvement. Proceedings of the Computer Vision and Pattern Recognition.

[39] Wang Z., Jin L., Wang S., Xu H. (2022). Apple stem/calyx real-time recognition using YOLO-v5 algorithm for fruit automatic loading system. Postharvest Biol. Technol..

[40] Li C., Li L., Jiang H., Weng K., Geng Y., Li L., Ke Z., Li Q., Cheng M., Nie W. (2022). YOLOv6: A single-stage object detection framework for industrial applications. arXiv.

[41] He Y., Sahma A., He X., Wu R., Zhang R. (2024). FireNet: A Lightweight and Efficient Multi-Scenario Fire Object Detector. Remote Sens..

[42] Wang C., Yeh I., Liao H.Y. (2024). Yolov9: Learning what you want to learn using programmable gradient information. arXiv.

[43] Wang A., Chen H., Liu L., Chen K., Lin Z., Han J., Ding G. (2024). Yolov10: Real-time end-to-end object detection. arXiv.

[44] Jocher G., Qiu J. (2024). Ultralytics YOLO11: Version 11.0.0[EB/OL]. https://github.com/ultralytics/ultralytics.

[45] Ren S., He K., Girshick R., Sun J. (2016). Faster R-CNN: Towards real-time object detection with region proposal networks. IEEE Trans. Pattern Anal. Mach. Intell..

[46] Li X., Lv C., Wang W., Li G., Yang L., Yang J. (2022). Generalized focal loss: Towards efficient representation learning for dense object detection. IEEE Trans. Pattern Anal. Mach. Intell..

[47] Lyu C., Zhang W., Huang H., Zhou Y., Wang Y., Liu Y., Zhang S., Chen K. (2022). Rtmdet: An empirical study of designing real-time object detectors. arXiv.

[48] Song C.-Y., Zhang F., Li J.-S., Xie J.-Y., Yang C., Zhou H., Zhang J.-X. (2023). Detection of maize tassels for UAV remote sensing image with an improved YOLOX model. J. Integr. Agric..

[49] Feng C., Zhong Y., Gao Y., Scott M.R., Huang W. Tood: Task-aligned one-stage object detection. Proceedings of the 2021 IEEE/CVF International Conference on Computer Vision (ICCV), IEEE Computer Society.

